# Traditional Herbal Formula Oyaksungi-San Inhibits Adipogenesis in 3T3-L1 Adipocytes

**DOI:** 10.1155/2015/949461

**Published:** 2015-02-23

**Authors:** Sae-Rom Yoo, Chang-Seob Seo, Hyeun-Kyoo Shin, Soo-Jin Jeong

**Affiliations:** Herbal Medicine Formulation Research Group, Herbal Medicine Research Division, Korea Institute of Oriental Medicine, 1672 Yuseongdae-ro, Yuseong-gu, Daejeon 305-811, Republic of Korea

## Abstract

*Background*. Oyaksungi-san (OYSGS) is a herbal formula that has been used for treating cardiovascular diseases in traditional Asian medicine. Here, we investigated the antiadipogenic effect of OYSGS extract in 3T3-L1 adipose cells. *Methods*. 3T3-L1 preadipocytes were differentiated into adipocytes with or without OYSGS. After differentiation, we measured Oil Red O staining, glycerol-3-phosphate dehydrogenase (GPDH) activity, leptin production, mRNA, and protein levels of adipogenesis-related factors. *Results*. OYSGS extract dramatically inhibited intracellular lipid accumulation in the differentiated adipocytes. It also significantly suppressed the (GPDH) activity, triglyceride (TG) content, and leptin production by reducing the expression of adipogenesis-related genes including lipoprotein lipase, fatty acid binding protein 4, CCAAT/enhancer-binding protein-alpha (C/EBP-*α*), and peroxisome proliferator-activated receptor gamma (PPAR-*γ*). Furthermore, OYSGS clearly enhanced phosphorylation of AMP-activated protein kinase (AMPK) as well as its substrate acetyl CoA (ACC) carboxylase. *Conclusions*. Our results demonstrate that OYSGS negatively controls TG accumulation in 3T3-L1 adipocytes. We suggest antiadipogenic activity of OYSGS and its potential benefit in preventing obesity.

## 1. Introduction

Excess energy intake and lack of activity lead to adipogenesis, which is a process of adipocyte differentiation in adipose tissue. Lipid-accumulated adipose tissue is a risk factor for several diseases leading to reduced life expectancy, such as type 2 diabetes, hyperlipidemia, cardiovascular disease, and cancers [1]. Past studies focused on antiobesity drugs using synthetic chemicals such as Orlistat or Sibutramine. However, because of their severe side effects [2, 3], there have been recent studies on antiobesity activities using natural products including herbal medicines or herbal medicinal products.

Several herbal formulas have been reported to have remarkable antiobesity effects* in vitro* and* in vivo*. For example, Hwangryunhaedok-tang (Orengedokuto) had inhibitory effects in 3T3-L1 cell adipogenesis [4], and Boiogito improved serum triglyceride contents in obese patients without different gene polymorphisms [5]. Another herbal formula, Bangpungtonse-oun-san (Bofu-tsusho-san), was shown to attenuate hyperlipidemia [6, 7] and body fat accumulation [8].

Oyaksungi-san (OYSGS; Wu yao shun qi san in China and Uyakujyunki-san in Japan) has been used for stroke, beriberi, and blood circulation disorders in Asian countries. It is a kind of decoction and consists of twelve herbs: Ephedrae Herba, Citri Unshius Pericarpium, Linderae Radix, Cnidii Rhizoma, Angelicae Dahuricae Radix, Batryticatus Bombyx, Citrus aurantium, Platycodonis Radix, Zingiberis Rhizoma, Zizyphi Fructus, Zingiberis Rhizoma Crudus, and Glycyrrhizae Radix et Rhizoma.

Several studies have reported that OYSGS has various biological effects against cancer [9], inflammation [10], and refractory trigeminal neuralgia [11]. In addition, OYSGS inhibited weight gain in high fat-fed rats [12], but the mechanism of action is not well understood. Therefore, we investigated the inhibitory effects of OYSGS on adipocyte differentiation and on lipid metabolism-related gene expression* in vitro* using 3T3-L1 cell line.

## 2. Materials and Methods

### 2.1. Preparation of OYSGS

Each of the 12 herbal components of OYSGS was mixed as listed in Table 1. For preparation of OYSGS water extraction, we used total 10.0 kg of dried herbs, which is 216.8-fold amount of single dose of OYSGS (10.0 kg; 46.125 g × 216.8) and is extracted in a 10-fold volume of water at 100°C for 2 h under pressure (1 kgf/cm^2^) using an electric extractor (COSMOS-660; Kyungseo Machine Co., Incheon, Korea). The water extract was then filtered through a standard sieve (number 270, 53 *μ*m; Chung Gye Sang Gong Sa, Seoul, Korea) and the solution was freeze-dried to a powder (Innova U725 Upright Freezer, Eppendorf, Hamburg, Germany). The yield of OYSGS in the water extract was 24.4% (2.4 kg). The extract was stored below 4°C. Voucher specimens (2008-KE27-1-12) have been deposited at the Herbal Medicine Formulation Research Group, Korea Institute of Oriental Medicine.

### 2.2. Chemicals and Reagents

Liquiritin (purity ≥ 95.0%), ferulic acid (purity ≥ 98.0%), and glycyrrhizin (purity ≥ 98.0%) were purchased from Wako Pure Chemicals (Osaka, Japan). Hesperidin (purity ≥ 98.0%) and neohesperidin (purity ≥ 98.0%) were purchased from Chengdu Biopurify Phytochemicals (Chengdu, China). Naringin (purity ≥ 98.0%) was purchased from Sigma-Aldrich (St. Louis, MO, USA). High performance liquid chromatography (HPLC) grade methanol, acetonitrile, and water were obtained from J. T. Baker (Phillipsburg, NJ, USA). Analytical reagent grade glacial acetic acid was purchased from Junsei (Tokyo, Japan).

### 2.3. Chromatographic Apparatus and Conditions

Chromatographic analysis was performed using the Shimadzu Prominence LC-20A series (Shimadzu, Kyoto, Japan) consisting of a solvent delivery unit (LC-20AT), an online degasser (DGU-20A3), a column oven (CTO-20A), an auto sample injector (SIL-20AC), and a photodiode array (PDA) detector (SPD-M20A). The data were acquired and processed using LC solution software (Version 1.24, Shimadzu, Kyoto, Japan). The six standard compounds (listed below) were separated on a Phenomenex Gemini C18 column (250 mm × 4.6 mm, 5 *μ*m, Torrance, CA, USA). The gradient elution of two mobile phase systems with 1.0% (v/v) acetic acid in water (A) and 1.0% (v/v) acetic acid in acetonitrile (B) was as follows: 15–65% B for 0–40 min, 65–100% B for 40–45 min, and 100% B for 45–50 min. The reequilibrium time was 10 min. The flow rate was kept constant at 1.0 mL/min, column temperature was maintained at 40°C, and injection volume was 10 *μ*L. The wavelength range of the PDA was 190–400 nm and detected wavelength was monitored at 254, 280, and 320 nm.

### 2.4. Preparation of Standard and Sample Solutions 

Stock solution of the six standard compounds—liquiritin, ferulic acid, naringin, hesperidin, neohesperidin, and glycyrrhizin—was dissolved in methanol at 1.0 mg/mL and kept below 4°C. For HPLC analysis, 400 mg of lyophilized OYSGS extract was dissolved in 20 mL of distilled water and then the solution was filtered through a SmartPor GHP 0.2 *μ*m syringe filter (Woongki Science, Seoul, Korea) before injection into the HPLC system.

### 2.5. Cell Culture and Adipocyte Differentiation

3T3-L1 preadipocytes were purchased from the American Type Culture Collection (Manassas, VA, USA) and maintained in Dulbecco's modified Eagle's medium supplemented with 10% normal calf serum (Zen-Bio Inc., Durham, NC, USA). To induce adipocyte differentiation, the 3T3-L1 cells were cultured to confluency. For adipocyte differentiation, confluent cells were maintained in 3T3-L1 differentiation medium containing 1 *μ*M dexamethasone, 1 *μ*g/mL insulin, and 0.5 mM 3-isobutyl-1-methylxanthine (Zen-Bio Inc., Durham, NC, USA) and fetal bovine serum for 2 days. The 10% FBS medium was replaced with fresh normal medium only containing 1 *μ*g/mL insulin for 2 days and with fresh 10% FBS medium without insulin for another 4 days. GW9662, a potent peroxisome proliferator-activated receptor gamma (PPAR-*γ*) antagonist, was used as a positive control (20 *μ*M).

### 2.6. Cytotoxicity Assay

Cell viability was assessed using the cell counting kit (CCK-8) (Dojindo Lab, Tokyo, Japan). Briefly, cells were seeded onto 96-well plates at a density of 4 × 10^3^ cells/well and induced to undergo adipocyte differentiation with exposure to OYSGS. After finishing the differentiation, CCK-8 solution (10 *μ*L/well) was added and incubated at 37°C for 4 h. Optical density (OD) was determined at wavelength of 450 nm using a microplate reader (Benchmark Plus Microplate Spectrophotometer, Bio-Rad Laboratories, Hercules, CA). Cell viability was calculated as the percentage of viable cells in the OYSGS-treated group versus untreated controls.

### 2.7. Oil Red O (ORO) Staining

The differentiated 3T3-L1 adipocytes were fixed with 10% formalin, washed with 70% ethanol and phosphate-buffered saline (PBS), and finally stained with ORO solution. Stained lipid droplets were visualized using an Olympus CKX41 inverted microscopy (Olympus, Tokyo, Japan). We calculated the number of ORO stained lipid droplets for each group using Metamorph offline (Molecular Devices Co., Sunnyvale, CA). Cells were exposed to OYSGS for 8 days during adipocyte differentiation.

### 2.8. Leptin Immunoassay

Leptin production was measured using mouse leptin quantification kit (R&D Systems, Minneapolis, MN, USA). The culture supernatants from differentiated adipocytes were added into 96-well polystyrene microplates coated with a polyclonal anti-leptin antibody for 2 h and then reacted with a polyclonal antibody against mouse leptin conjugated to horseradish peroxidase (HRP) for 2 h. The substrate solution was added to each well and incubated for 30 min at room temperature. Adding a stop solution terminated the reaction and the OD was read using a microplate reader as above at 450 nm with a reference wavelength of 540 nm.

### 2.9. Triglyceride (TG) Colorimetric Assay

TG content was determined using Triglyceride Quantification kit (Bio Vision, Mountain View, CA, USA). Cells were homogenized in TG assay buffer containing 5% noniodet p-40, slowly heated to 80°C for 5 min, and centrifuged for 2 min to remove insoluble materials. The samples were mixed with lipase and a TG reaction mixture containing a TG probe and incubated at room temperature for 1 h. Sample absorbance was measured at 570 nm.

### 2.10. Glycerol-3-Phosphate Dehydrogenase (GPDH) Activity Assay

GPDH activity was analyzed using GPDH activity assay kit (TaKaRa Bio Inc., Tokyo, Japan) according to the manufacturer's instructions. In brief, cells were washed twice with PBS, lysed using the enzyme extraction buffer, and centrifuged at 10,000 rpm for 5 min at 4°C. The supernatants were mixed with the substrate solution at 30°C and the decrease in absorbance was determined at 340 nm to calculate the change in absorbance per minute (ΔOD_340_) using a microplate reader as above. GPDH enzyme activity was calculated from the following manufacturer's instructions manual.

### 2.11. RT-PCR

Total RNA was extracted using Trizol reagent (Invitrogen Life sciences, Carlsbad, CA, USA) according to the manufacturer's instructions. cDNA was synthesized from 1 *μ*g of total RNA using iScript cDNA synthesis kit (Bio-Rad Laboratories, Hercules, CA, USA) and subjected to PCR reactions with rTaq DNA polymerase (ELPIS Biotech Inc., Daejeon, South Korea). All primer sequences are shown in Table 2. The PCR conditions were 22–28 cycles of 94°C for 30 sec, 50–60°C for 1 min, and 72°C for 1.5 min. The amplification products were then separated by electrophoresis on 1% agarose gels and detected with a Molecular Imager Gel Doc XR System (Bio-Rad Laboratories, Hercules, CA, USA).

### 2.12. Western Blot Analysis

Cells were lysed in lysis buffer containing protease inhibitors (Roche Applied Science, Indianapolis, IN, USA). The lysates were centrifuged at 14,000 g for 15 min at 4°C, and the protein concentrations in the supernatants were determined using Bradford reagent (Bio-Rad Laboratories, Hercules, CA). The proteins were subjected to western blotting on precast gels (Bio-Rad Laboratories, Hercules, CA, USA) and transferred to polyvinylidene difluoride membranes (Amersham Biosciences, Piscataway, NJ, USA). After nonspecific binding sites were blocked with 5% (w/v) nonfat dry milk dissolved in TBST buffer (10 mM Tris-HCl pH 7.5, 150 mM NaCl, 0.1% Tween 20), the membranes were incubated with primary antibodies anti-phospho-AMP-activated protein kinase (AMPK), AMPK (Cell Signaling Technology, Danvers, MA, USA), phospho-acetyl CoA carboxylase (ACC), ACC (Millipore, Billerica, MA), and *β*-actin (Santa Cruz Biotechnology, Santa Cruz, CA, USA). After the removal of the primary antibody, the membranes were washed three times with TBST buffer at room temperature and incubated with HRP-conjugated secondary antibody (Immunoresearch, West Grove, PA, USA) for 1 h at room temperature. The membranes were rewashed with TBST buffer, and the immunoreactive bands were visualized with ECL reagent (Thermo Scientific, Rockford, IL, USA).

### 2.13. Statistical Analysis

Data are expressed as the means ± standard error of the mean (SEM). Statistical significance was determined using one-way analysis of variance for independent means, in the GraphPad InStat ver. 3.10 program (Graphpad Software, Inc., San Diego, CA, USA). Significance was set at *P* < 0.05.

## 3. Results

### 3.1. HPLC Analysis of OYSGS

The calibration curves of all analytes were calculated by plotting the peak areas (*y*) versus the corresponding concentrations (*x*, *μ*g/mL) using the standard solutions. The tested concentration ranges were as follows: liquiritin, ferulic acid, and glycyrrhizin: 0.39–50.00 *μ*g/mL, naringin: 3.13–400.00 *μ*g/mL, hesperidin: 1.95–250.00 *μ*g/mL, and neohesperidin: 1.56–200.00 *μ*g/mL. The correlation coefficients (*r* ^2^) of the six compounds showed good linearity over the ranges at ≥0.9998. The limit of detection and the limit of quantification ranges of the six tested compounds were 0.02–0.09 and 0.06–0.29 *μ*g/mL. These results are summarized in Table 3.

**Figure 1:**
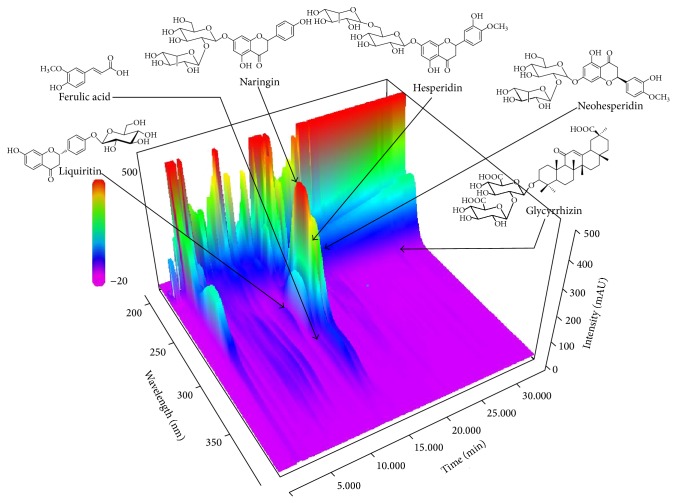
Three-dimensional chromatogram of OYSGS by HPLC-PDA.

Using optimized chromatography conditions, three-dimensional chromatogram was obtained using HPLC-PDA detector. The typical three-dimensional HPLC chromatogram of OYSGS is shown in Figure 1. The contents of the six marker compounds, liquiritin, ferulic acid, naringin, hesperidin, neohesperidin, and glycyrrhizin, in OYSGS were 0.86, 0.33, 9.19, 5.96, and 5.65 mg/g, respectively (Table 4).

### 3.2. OYSGS Had No Cytotoxicity for 3T3-L1 Preadipocytes or Differentiated Adipocytes

**Figure 2:**
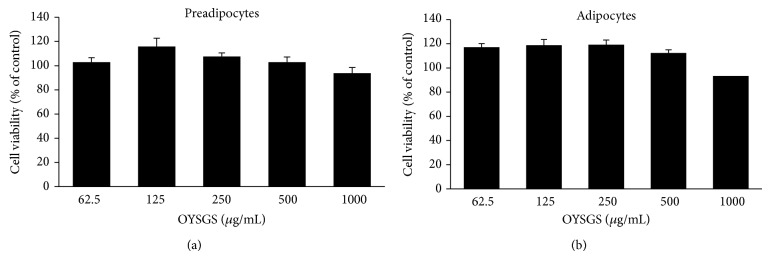
Cytotoxicity of OYSGS extract in 3T3-L1 cells. The effect of OYSGS extract on cell viability was evaluated in 3T3-L1 preadipocytes (a) and adipocytes (b) by CCK-8 assay. Cells were treated with various concentrations of OYSGS. Results are presented as the mean ± SEM. The graph represents the cytotoxicity from two independent experiments.

To evaluate the possible cytotoxicity of OYSGS against 3T3-L1 preadipocytes, the cells were treated with various concentrations of OYSGS (62.5, 125, 250, 500, or 1000 *μ*g/mL) for 24 h. As shown in Figure 2(a), OYSGS had no significant effect on the viability of 3T3-L1 cells. In addition, the cytotoxicity of OYSGS in differentiated adipocytes was assessed in 3T3-L1 cells induced to differentiate into adipocytes. During differentiation for 8 days, the cells were exposed to various concentrations of OYSGS (62.5, 125, 250, 500, or 1000  *μ*g/mL). The cell viability was maintained at over 93% even at 1000 *μ*g/mL (Figure 2(b)). Thus, there was no toxic effect of OYSGS on preadipocytes or adipocytes.

### 3.3. OYSGS Inhibited Adipogenesis in 3T3-L1 Adipocytes

**Figure 3:**
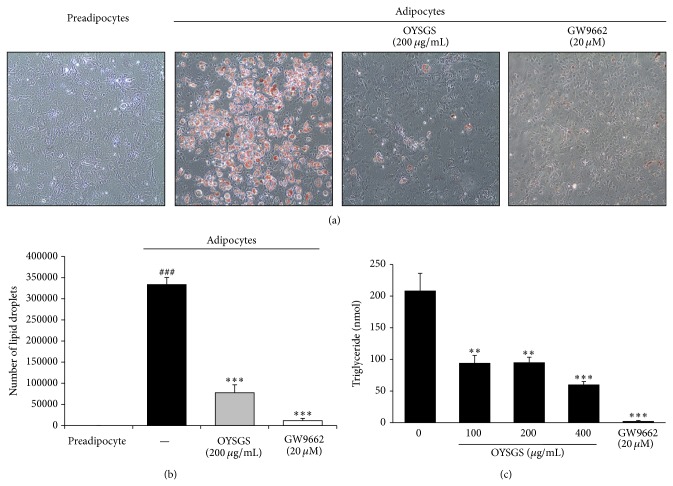
Inhibitory effects of OYSGS extract on lipid accumulation in 3T3-L1 adipocytes. Adipocyte differentiation was induced for 8 days with or without OYSGS. (a) After the differentiation, lipid accumulation in the cells was stained with Oil Red O dye and visualized under an inverted microscope at 200x of magnification. (b) Lipid droplets were quantified using Metamorph offline (Molecular Devices Co., Sunnyvale, CA). (c) The triglyceride contents were measured enzymatically using a commercial kit at 570 nm. Results are presented as the mean ± SEM. ^###^ *P* < 0.001 compared with the undifferentiated group. ^**^ *P* < 0.01 and ^***^ *P* < 0.001 compared with the differentiated group. The graph represents the number of lipid droplets (b) and TG contents (c) from three independent experiments.

Lipid accumulation in adipocytes is one hallmark of adipogenesis [13]. ORO staining was performed to determine whether OYSGS could influence lipid accumulation in adipose cells. As shown in Figure 3(a), the content of lipid droplets detectable with ORO staining was markedly increased in adipocytes compared with preadipocytes. Quantification of lipid droplets was further confirmed inhibitory effect of OYSGS on lipid accumulation (Figure 3(b)). Additionally, lipid droplets contain TG, an important metabolic factor in obesity. Thus, we assessed change in the contents of TG in OYSGS-treated adipocytes. Consistent with the results of ORO assay, a significant reduction of TG contents was observed in OYSGS-treated adipocytes compared with differentiated control cells (Figure 3(c)). Similarly, GW9662, a positive control, dramatically reduced lipid accumulation in adipocytes (Figure 3).

**Figure 4:**
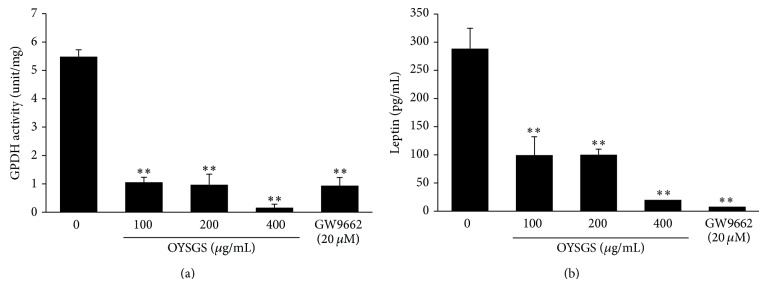
Inhibitory effects of OYSGS extract on the productions of leptin in 3T3-L1 adipocytes. Cell differentiation was induced for 8 days with or without OYSGS. (a) Cell lysates were prepared and used for analyzing the GPDH activity. (b) Leptin concentration in the supernatant was measured using a leptin quantification kit. Results are presented as the mean ± SEM. ^**^ *P* < 0.01 and ^***^ *P* < 0.001 compared with the differentiated group. The graph represents the GPDH activity (a) and leptin production (b) from three independent experiments.

GPDH is an enzyme that generates glycerol-3-phosphate from dihydroxyacetone phosphate in adipocytes for lipid biosynthesis [14]. Therefore, the enzymatic activity of GPDH was measured in 3T3-L1 adipocytes treated with various concentrations of OYSGS. GPDH activity (Figure 4(a)) was significantly reduced in OYSGS-treated cells compared with untreated controls. Consistent with results of the GPDH and TG assay, leptin concentration decreased in a dose-dependent manner (Figure 4(b)).

### 3.4. OYSGS Suppressed the Expression of Adipogenesis-Related Biomarkers at the mRNA and Protein Levels

**Figure 5:**
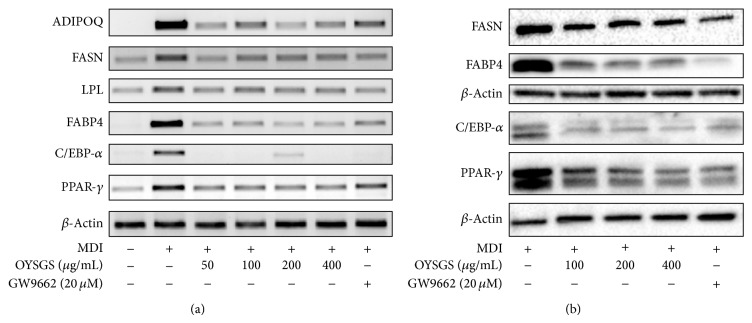
Effects of OYSGS extract on lipid metabolism-related gene and protein expression levels in 3T3-L1 adipocytes. Preadipocytes were differentiated into adipose cells by adding differentiation medium for 6 days. Various concentrations of OYSGS were added during this. After differentiation, we analyzed (a) mRNA and (b) protein expression levels in adipose cells.

Adipogenesis is accompanied by alterations in the expression of various transcriptional factors and adipogenesis-specific markers [15, 16]. As shown in Figure 5(a), the mRNA PPAR-*γ* and CCAAT/enhancer binding protein-alpha (C/EBP-*α*), major transcriptional factors, as well as expressions of PPAR-*γ* target genes, including those encoding fatty acid binding protein 4 (FABP4), lipoprotein lipase (LPL), fatty acid synthase (FASN), and adiponectin (ADIPOQ) in the adipogenesis pathway, were significantly increased in differentiated cells. In contrast, OYSGS treatment effectively reduced the mRNA levels of PPAR-*γ* and C/EBP-*α*. Furthermore, OYSGS treatment led to the suppression of PPAR-*γ* target gene expression levels, including FABP4, LPL, FASN, and ADIPOQ in 3T3-L1 adipocytes.

Consistent with the RT-PCR results, protein expressions of PPAR-*γ*, C/EBP-*α*, FABP4, and FASN were decreased by OYSGS treatment compared with differentiated control cells (Figure 5(b)). As we expected, GW9662 blocked PPAR-*γ* and C/EBP-*α*-related mRNA and protein expression levels compared with differentiated cells.

### 3.5. OYSGS Regulated AMPK/ACC Signaling in 3T3-L1 Adipocytes

**Figure 6:**
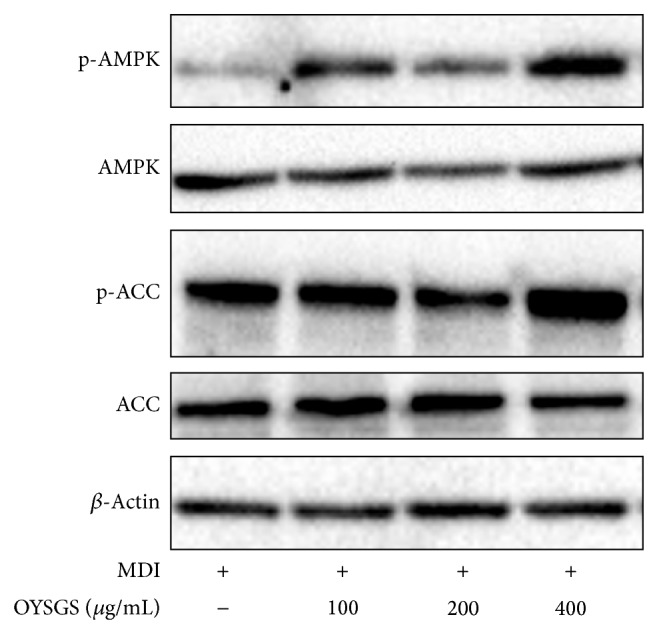
Effects of OYSGS extract on the AMPK/ACC signaling in 3T3-L1 adipocytes. Preadipocytes were differentiated into adipocytes by adding differentiation medium with or without OYSGS. Cell lysates were prepared from the differentiated cells and subjected to western blotting for p-AMPK, p-ACC, and *β*-actin.

AMPK plays a sensor for cellular energy regulation, which is activated in response to increases in the AMP : ATP ratio [17]. OYSGS treatment remarkably enhanced the level of phosphorylated AMPK (p-AMPK) at 400 *μ*g/mL of treatment in 3T3-L1 adipocytes. OYSGS also increased the level of phosphorylated ACC (p-ACC), a substrate of AMPK, in the differentiated cells (Figure 6), indicating a critical role for AMPK/ACC signaling during the inhibition of adipogenesis in OYSGS. As the p-AMPK/ACC ratio was increased, the total protein level of AMPK and ACC remained constant.

## 4. Discussion

“Herbalism” is the study and use of the medicinal properties of herbs, phytochemicals, and herbal mixtures. Traditional herbal medicine has been traditionally used for treating immunologic disorders including the common cold, asthma, and allergies in Eastern countries including Korea, Japan, China, and India. It has increased in popularity in the past two decades among patients seeking alternative treatments to conventional Western allopathic medicine. Traditional herbal medicine focuses on restoring a “yin-yang” balance rather than treating a particular disease or medicinal condition. A clinical herbalist can combine several kinds of herbs based on a diagnosis, using herbal formula, which can be modified to fit each individual's complaint and constitution.

OYSGS was first described in the Song dynasty (China, AD1107) and has been used for treatment of vascular related disorders. A new approach for using OYSGS has been studied for several decades; recent studies indicated that OYSGS has diverse effect on several diseases such as cancer [9] and inflammation [10]. One of these researches, Jung and Lee, reported that OYSGS treatment for 8 weeks significantly reduced the onset of obesity in Sprague Dawley rats [12]. However the molecular mechanisms of the antiobesity activity of OYSGS* in vitro* or* in vivo* remain to be established.

Among the 12 OYSGS components, the antiobesity effects of three herbs, Citrus aurantium, Platycodonis Radix, and Citri Unshius Pericarpium, have been reported. Citrus aurantium [18] and Platycodonis Radix [19] have attenuated weight gain in obese mouse via AMPK pathway. Citri Unshius Pericarpium also inhibits the differentiation of 3T3-L1 adipocytes [20]. In the present study, we investigated the inhibitory effects of OYSGS on adipogenesis—an important process associated with obesity—and the regulatory molecular mechanisms using 3T3-L1 adipocytes.

Prior to testing the antiobesity effect of OYSGS, we needed to verify this formula. Based on the Korean Pharmacopoeia [21], an optimized HPLC-PDA method was applied for simultaneous determination of the six marker compounds in OYSGS. The components investigated were naringin, hesperidin, and neohesperidin from Citri Unshius Pericarpium and Aurantii Fructus Immaturus, ferulic acid from Cnidii Rhizoma, and liquiritin and glycyrrhizin from Glycyrrhizae Radix et Rhizoma. Among these components, flavonoids such as naringin, hesperidin, and neohesperidin, which are marker components of Citri Unshius Pericarpium and Aurantii Fructus Immaturus, were detected at levels of 9.19, 5.96, and 5.65 mg/g, respectively, as the main compounds compared with the others in the OYSGS extract. These results could be valuable for the efficient quality control of OYSGS preparations.

The 3T3-L1 preadipocytes were differentiated into mature adipocytes with or without OYSGS. Interestingly, the number of lipid droplets, fat storage organelles, was markedly decreased in the OYSGS treatment group compared with the differentiated group (Figure 3(a)). Fat accumulation in adipose tissue occurs at a late stage in adipogenesis and is associated with increased TG concentrations [22] and adipose tissue mass [23]. For this reason, we assessed whether OYSGS exerted any effect on TG related markers. Consistent with ORO staining results, OYSGS significantly reduced intracellular TG contents (Figure 3). GPDH activity, the first step in TG synthesis in mature adipocytes, was significantly decreased by OYSGS treatment (Figure 4) [24].

Adipose tissue acts as an endocrine organ involved in energy homeostasis and inflammation. Leptin, an adipose tissue-specific adipokine, links body fat storage to adaptive responses in the key control of energy balance [25]. Intra- and extracellular protein levels of leptin are highly correlated with body fat mass and adipocyte size [26]. In our study, leptin levels were markedly reduced following OYSGS treatment. These results suggest a biological property of OYSGS as a negative regulator of adipogenesis.

Adipocyte differentiation is a process that is tightly controlled by molecular and cellular mechanisms, including transcriptional factors and extracellular proteins [14, 15]. The main transcriptional factors are PPAR-*γ* and C/EBP-*α*.  PPAR-*γ* is an indispensable factor for adipocyte differentiation [27] and is involved in a positive feedback loop with C/EBP-*α* to sustain expression mutually [28]. In this study, OYSGS considerably reduced the mRNA and protein expression levels of PPAR-*γ* and C/EBP-*α* in 3T3-L1 adipocytes (Figure 4). The expressions of PPAR-*γ* and C/EBP-*α* together regulate the downstream target genes AdipoQ, LPL, and FABP4 [29, 30]. Consistent with mRNA expression of PPAR-*γ* and C/EBP-*α*, those of AdipoQ, LPL, and FABP4 were also decreased in the OYSGS treatment group (Figure 5). Furthermore, the expression level of FASN, which encodes a rate-limiting enzyme that catalyzes the formation of long-chain fatty acids from acetyl-coA [31], appeared to be reduced by the OYSGS treatment (Figure 5).

We hypothesized that a reduction in TG accumulation by the OYSGS treatment in part mediated fatty acid synthesis. To test our hypothesis, we investigated the effect of OYSGS on the activation of AMPK, a sensor for cellular energy regulation, which is activated in response to an increase in the AMP : ATP ratio. AMPK activation can inhibit adipocyte differentiation [32] and modulates the transcription of many genes involved in energy metabolism including lipogenesis, TG synthesis [33, 34], and fatty acid oxidation [35]. Several studies have reported a critical role of AMPK in the antiadipogenic action of natural products including herbal formula, herbal plants, and phytochemicals. For instance, the Jinqi formula inhibits TG accumulation via activation of AMPK [36]. Another herbal formula, Qushi Huayu, decreases hepatic lipid accumulation by stimulating AMPK phosphorylation* in vivo* and* in vitro* [37]. In the present study, we found that OYSGS enhanced phosphorylation of AMPK as well as its substrate ACC, which is the rate-limiting enzyme in fatty acid synthesis (Figure 6).

In conclusion, our results have demonstrated that OYSGS inhibited adipogenesis in 3T3-L1 adipocytes as indicated by a significant reduction in TG accumulation without cytotoxicity. Furthermore, these suppressive effects of OYSGS are possibly mediated by downregulated expression of adipogenesis-related genes. Thus, OYSGS might act as a therapeutic agent for preventing obesity.

## Figures and Tables

**Table 1:** Crude composition of Oyaksungi-san.

Local name	Herbal name	Scientific name	Amount (g)	Location of origin
Ma-huang	Ephedrae Herba	*Ephedra sinic*a Stapf	5.625	China
Jin-pi	Citri Unshius Pericarpium	*Citrus unshiu* (Yu. Tanaka ex Swingle) Marcow.	5.625	Jeju, Korea
O-yak	Linderae Radix	*Lindera aggregate* (Sims) Kosterm.	5.625	China
Chun-gung	Cnidii Rhizoma	*Cnidium officinale* Makino	3.750	Yeongcheon, Korea
Baek-ji	Angelicae Dahuricae Radix	*Angelica dahurica* (Hoffm.) Benth. & Hook.f. ex Franch. & Sav.	3.750	Yeongcheon, Korea
Baek-jang-gam	Batryticatus Bombyx	*Bombyx mori* L./*Beauveria bassiana* (Bals.) vuill.	3.750	China
Ji-gak	Citrus aurantium	*Citrus* ×* aurantium* L.	3.750	China
Gil-gyeong	Platycodonis Radix	*Platycodon grandiflorus* (Jacq.) A. DC.	3.750	Yeongcheon, Korea
Geon-gang	Zingiberis Rhizoma	*Zingiber officinale* Roscoe	3.750	Yeongcheon, Korea
Dae-jo	Zizyphi Fructus	*Ziziphus jujuba* Mill.	3.750	Yeongcheon, Korea
Saeng-gang	Zingiberis Rhizoma Crudus	*Zingiber officinale* Roscoe	1.875	Yeongcheon, Korea
Gam-cho	Glycyrrhizae Radix et Rhizoma	*Glycyrrhiza uralensis* Fisch.	1.125	China

	Net amount (g)		46.125	

**Table 2:** Primer sequences for RT-PCR.

Gene		Primer sequence
ADIPOQ	Forward	GGGTGAGACAGGAGATGTTGGAATG
	Reverse	GCCAGTAAATGTAGAGTCGTTGACG

FASN	Forward	CAGTATAAGCCCAAGGCCAA
	Reverse	TAGCCCTCCCGTACACTCAC

LPL	Forward	CTGCTGGCGTAGCAGGAAGT
	Reverse	GCTGGAAAGTGCCTCCATTG

FABP	Forward	TGGAAGCTTGTCTCCAGTGA
	Reverse	ATTTCCATCCAGGCCTCTT

C/EBP-*α*	Forward	ATCCCAGAGGGACTGGAGTT
	Reverse	AAGTCTTAGCCGGAGGAAGC

PPAR-*γ*	Forward	TATGGAGTTCATGCTTGTGA
	Reverse	CGGGAAGGACTTTATGTATG

*β*-Actin	Forward	AATGTAGTTTCATGGATGCC
	Reverse	CCAGATCATGTTTGAGACCT

**Table 3:** Regression equations, linearity, LOD, and LOQ for six compounds.

Analyte	Linear range (*μ*g/mL)	Regression equation^a^	Correlation coefficient (*r* ^2^)	LOD^b^ (*μ*g/mL)	LOQ^c^ (*μ*g/mL)
Liquiritin	0.39–50.00	*y* = 16085.82*x* + 3886.14	0.9998	0.08	0.28
Ferulic acid	0.39–50.00	*y* = 46969.88*x* − 9733.35	0.9998	0.02	0.06
Naringin	3.13–400.00	*y* = 16249.71*x* + 46309.18	0.9998	0.07	0.24
Hesperidin	1.95–250.00	*y* = 17380.77*x* + 30132.79	0.9999	0.07	0.25
Neohesperidin	1.56–200.00	*y* = 20898.85*x* + 29610.91	0.9998	0.06	0.19
Glycyrrhizin	0.39–50.00	*y* = 8294.45*x* + 2548.76	0.9999	0.09	0.29

^a^ *y*: peak area (mAU) of compounds; *x*: concentration (*μ*g/mL) of compounds.

^b^LOD = 3 × signal-to-noise ratio.

^c^LOQ = 10 × signal-to-noise ratio.

**Table 4:** The amount (mg/g) of six marker compounds in Oyaksungi-san (*n* = 3).

Analyte	Mean	SD	RSD (%)	Source
Liquiritin	0.86	0.01	1.46	GRR^a^
Ferulic acid	0.33	0.01	2.07	CR^b^
Naringin	9.19	0.04	0.43	CUP^c^, AFI^d^
Hesperidin	5.96	0.08	1.27	CUP, AFI
Neohesperidin	5.65	0.02	0.43	CUP, AFI
Glycyrrhizin	0.93	0.02	2.43	GRR

^a^Glycyrrhizae Radix et Rhizoma.

^b^Cnidii Rhizoma.

^c^Citri Unshius Pericarpium.

^d^Aurantii Fructus Immaturus.
